# Erianin alleviates collagen-induced arthritis in mice by inhibiting Th17 cell differentiation

**DOI:** 10.1515/biol-2022-0703

**Published:** 2023-09-04

**Authors:** Sen-Wei Tsai, Jou-Hsuan Wang, Yu-Kang Chang, Chi-Chen Lin

**Affiliations:** Department of Physical Medicine and Rehabilitation, Taichung Tzu Chi Hospital, Buddhist Tzu Chi Medical Foundation, Taichung 427, Taiwan; School of Medicine, Tzu Chi University, Hualien 970, Taiwan; Department of Medical Research, Tungs’ Taichung MetroHarbor Hospital, Taichung 435, Taiwan; Institute of Biomedical Science, National Chung Hsing University, Taichung, Taichung 402, Taiwan; Department of Medical Research, China Medical University Hospital, Taichung 404, Taiwan; Department of Medical Research, Taichung Veterans General Hospital, Taichung 407, Taiwan; Department of Pharmacology, College of Medicine, Kaohsiung Medical University, Kaohsiung 807, Taiwan; Department of Post-Baccalaureate Medicine, College of Medicine, National Chung Hsing University, Taichung 402, Taiwan; Department of Nursing, Jen-Teh Junior College of Medicine, Nursing and Management, Miaoli 356, Taiwan

**Keywords:** rheumatoid arthritis, collagen-induced arthritis, Th17 cell, erianin, natural compound, inflammation

## Abstract

Rheumatoid arthritis (RA) is a chronic autoimmune disorder. Its pathogenesis is complicated but highly related to aberrant Th17 overactivation. Uncontrolled Th17 cell expansion and activation in populations and associated activities contribute to the progression of RA. Although clinical RA remedies are available, not all RA patients respond to these treatments, and adverse effects are always a concerning issue during treatment. To expand the repertoire of possible anti-RA remedies, we chose the phytochemical compound erianin, isolated from *Dendrobium* sp., and evaluated its antiarthritic effect *in vitro* and *in vivo*. We found that erianin efficiently controlled the differentiation and activation of Th17 cell development from primary CD4 T cells, limiting IL-17A cytokine production and RORγT transcript generation. In line with molecular docking models, the essential signaling pathway for Th17 polarization, the JAK/STAT3 pathway, was inhibited upon erianin treatment, with dose-dependent inhibition of phosphorylation shown by western blotting. More importantly, erianin treatment reduced arthritic manifestations and proinflammatory cytokine levels in collagen-induced arthritis (CIA) mice, as well as protecting the joint histological microstructure. Overall, erianin revealed a promising inhibitory effect on Th17 overactivation and decreased disability in CIA mice. Therefore, erianin could be further developed as a candidate RA remedy.

## 1 Introduction

Over the last two decades, Th17 regulation in the pathogenesis of rheumatoid arthritis (RA), a well-known chronic inflammatory illness, has received increasing attention [1]. The association between Th17 activation and the severity of RA has been shown in more and more data, demonstrating the need to control Th17 activity while treating RA patients [2]. Since the initial discovery of Th17 cells, our knowledge of their pathogenicity has advanced significantly. It has been demonstrated that cytokines like IL-17 and IL-22 released by Th17 cells can directly increase inflammation in the synovial joints and indirectly worsen the course of RA by increasing vesicular permeability and bone erosion, which are respectively mediated by endothelial cells and osteoblasts [3]. Th17 cells have subsequently become a therapeutic focus, and treatments that can modulate Th17 activity have also been found.

Due to its many bioactivities, *Dendrobium* sp. has long been utilized for therapeutic purposes [4]. Many pure compounds have been isolated from *Dendrobium* sp., including moscatilin from *Dendrobium moniliforme* [5] and confusarin and erianin from *Dendrobium chrysotoxum* [6]. Two-phenyl ring complex erianin (Eri), with numerous methoxyl substitutions, has been shown to primarily inhibit angiogenesis and cancer cell proliferation [7,8,9,10]. In a more recent publication, erianin was identified to induce ferroptosis via Ca^2+^/CaM signaling, rendering erianin a relatively promising prodrug [7]. In addition to the abovementioned activities, erianin is associated with inflammation [11] because it acts as an anti-inflammatory agent [12,13] and RA is also characterized by an increased burden of inflammation [14]. Therefore, studying erianin in an arthritis model is reasonable. That is why we aimed to clarify the anti-inflammatory activity of erianin under arthritic conditions and elucidate whether a Th17-modulating capability could be involved in the mechanism of action of erianin against arthritis because herbal medicine has attracted considerable attention, including the proposal of a wide variety of novel phytochemical candidates for RA therapy in the future.

To this end, we adapted and established a widely used arthritic model, the collagen-induced arthritics (CIA) mouse model, to verify the therapeutic effectiveness of erianin and explore the underlying mechanisms. We presented *in vivo* evidence demonstrating that erianin possessed comparable therapeutic effects to the current clinical RA drug methotrexate (MTX) and that erianin could profoundly ameliorate pathogenic Th17 activity.

## 2 Materials and methods

### 2.1 Th17 cell differentiation *in vitro*

CD4+ T cells were isolated from spleens harvested from female DBA1/1 mice using adverse magnetic bead-based selection and a mouse naïve CD4+ T-cell isolation kit (Miltenyi Biotec, CA, USA; Cat # 130-104-453) according to the manufacturer’s procedure. Cells were grown in RPMI 1640 medium supplemented with 10% fetal bovine serum (HyClone, Carlsbad, CA, USA), 100 U/ml penicillin, and 100 g streptomycin (all from Invitrogen-Gibco).

To stimulate Th17 cell differentiation, 1 µg/ml plate-bound anti-CD3, 1 µg/ml soluble anti-CD28, 50 ng/ml IL-6 (PeproTech), 10 ng/ml TGF-1 (PeproTech), 2 g/ml anti-IFN-γ antibody (clone R4-6A2, eBioscience), and 2 µg/ml anti-IL-4 antibody were added to cells to promote differentiation. IL-6/IL-23/TGF-β was added to each culture well, followed by erianin treatment (1 and 5 nM), incubation for 72 h, and determination of cytokine levels in the culture supernatant using ELISA.


**Ethical approval:** The research related to animal use has been complied with all the relevant national regulations and institutional policies for the care and use of animals and has been approved by the NCHU Committee for the Care and Use of Laboratory Animals (Approval No. 112027).

### 2.2 RNA isolation and real-time RT-PCR

TRIzol (Life Technologies, Grand Island, NY, USA) was used to extract total RNA from stimulated CD4+ T cells according to the manufacturer’s instructions. The Superscript III Reverse Transcriptase system was then used for cDNA reverse transcription (Invitrogen). RT-qPCR was carried out using the AB 7500 Fast System (Applied Biosystems, Foster City, CA) after the addition of SYBR Green Master Mix (Roche, Basel, Switzerland). The following primer sequences were used: mouse IL-17A, forward 5′-TTTTCAGCAAGGAATGTGGA-3′ and reverse 5′-TTCATTGTGGAGGGCAGAC-3′; mouse RORγt, forward 5′-CCGCTGAGAGGGCTTCAC-3′ and reverse 5′-TGCAGGAGTAGGCCACATTACA-3′; and mouse GAPDH, forward 5′-GGCATCCTGGGCTACACTGA-3′ and reverse 5′-GGAGTGGGTGTCGCTGTTG-3′.

### 2.3 Cytokine production measured by ELISA

T cells were cultured in a Th17 cell culture medium with or without erianin for 72 h, and then, the levels of cytokines, including TNF-α, IL-6, IL-17A, and IL-10, were measured by ELISA (ELISA eBioscience). The final concentration of each cytokine was normalized against the weight of the sample tissues before homogenization (50 mg of tissue/ml) in tissue lysis buffer (T-PER Tissue Protein Extraction Reagent, Thermo, USA) containing UltraCruz^®^ Protease Inhibitor Cocktail (Santa Cruz Biotechnology).

### 2.4 Flow cytometry

Under Th17-polarizing conditions, isolated T cells in 96-well plates were treated with or without erianin for 72 h and then stimulated with phorbol-12-myristate-13-acetate (PMA, 100 ng/ml)/ionomycin (500 ng/ml, Sigma-Aldrich, St. Louis, MO, USA) and GolgiStop (BD Bioscience, San Diego, CA, USA) for the last 4 h. CD4+ T cells were stained with a phycoerythrin (PE)-conjugated anti-CD4 antibody at 4°C for 20 min in the dark (eBioscience). The cells were fixed and permeabilized for intracellular staining using the Cytofix/Cytoperm Plus kit (BD Biosciences), labeled with a FITC-conjugated anti-IL-17A antibody, and incubated in the dark at room temperature for 20 min. The Th17 cell subset was identified as double positive for CD4 and IL-17A and quantified on an Accuri 5 flow cytometer to determine the mean fluorescence intensity.

Spleen cells were extracted using the Mouse Spleen Dissociation Kit (Miltenyi Biotec) and suspended in RPMI 1640 medium supplemented with 200 μl of 10% fetal calf serum, 50 g/ml gentamicin, 2 mM glutamine, and 50 M 2-mercaptoethanol. After 72 h of stimulation with bovine CII (50 g/ml), the cells underwent surface staining with a PerCP/Cyanine 5.5-conjugated anti-CD4 antibody (clone GK1.5; BioLegend, San Diego, CA), followed by intracellular staining for IL-17A and Foxp3 with FITC-labeled anti-IL-17A and PE-labeled anti-Foxp3 primary antibodies; the cells were analyzed on an Accuri C5 cytometer.

### 2.5 Western blot analysis

T cells under Th17-polarizing conditions were lysed with lysis buffer (Cell Signaling Technologies, Inc., Danvers, MA) following erianin treatment or no treatment, and total protein was extracted and quantified using BCA Protein Assay Reagent (Pierce). Anti-phosphorylated STAT3 (Cell Signaling Technology Inc.), anti-STAT3 (Cell Signaling Technology Inc.), anti-phosphorylated JAK2 (Cell Signaling Technology Inc.), anti-JAK2 (Cell Signaling Technology Inc.), and anti-GAPDH (Cell Signaling Technology Inc.) primary antibodies were incubated with cell lysates transferred to PVDF membranes at 4°C overnight. Following washing, the membranes were incubated with secondary antibodies conjugated with horseradish peroxidase (The Jackson Laboratory, Sacramento, CA) and developed by adding LumiFlash Ultima Chemiluminescent substrate (Visual protein, Taipei, Taiwan; LF08-500) to emit luminescence, which could be detected by a Hansor Luminescence Imaging System (Taichung, Taiwan). Band densities were evaluated using the National Institutes of Health application ImageJ 1.47 (Bethesda, MD, USA).

### 2.6 Animals and induction of CIA

Six- to eight-week-old female DBA/1J mice were purchased and housed in an SPF environment (23°C, 60% RH) with a defined light/dark cycle (12 h light/12 h dark). The NCHU Committee for the Care and Use of Laboratory Animals authorized all experimental techniques (Approval No. 112027).

For induction of CIA, mice were injected subcutaneously at the base of the tail with 100 μg of bovine CII (Chondrex, WA, USA; Cat# 20021) and 1 mg/ml complete Freund’s adjuvant (Sigma-Aldrich). CII emulsified in incomplete Freund’s adjuvant at a 1:1 ratio (v/v) was given to the mice for 21 consecutive days, followed by daily intragastric administration of erianin (5 mg/kg) dissolved in 95% olive oil and DMSO (v/v) from days 21 to 42. Vehicle-treated mice received a 95% olive oil, DMSO mixture instead of erianin in DMSO, and MTX administered via intraperitoneal injection was used as a positive control. When evaluating arthritic symptoms, arthritis scores (0–4) were rated by double-blinded observers [15] based on an index with a maximal score of 16 (4 points × 4 paws). The mice were anesthetized and sacrificed on day 43 to collect spleens and joint tissues. The spleens were used to isolate splenocytes, and the joint tissues were subjected to paraffin embedding and sectioning to examine histological alterations, including cell infiltration, cartilage breakdown, and bone erosion, which were scored and characterized as described in previous work [16].

### 2.7 Molecular docking

The protein structures of JAK2 (PDB ID: 7F7W) and STAT3 (PDB ID: 3CWG) were obtained from the Protein Database (PDB), and the structure of erianin was obtained from ChemSpider.com. Molecular docking was performed via Swissdock [17]. ICM-browser displayed the possible docking models and selected representative models based on lower Δ*G* values.

### 2.8 Statistical analysis

Data are presented as the means ± standard error of the mean (SEM). In the *in vitro* cell experiments, we analyzed the differences among all groups using one-way analysis of variance (ANOVA) with the post hoc Tukey HSD test. For the mouse body weight and arthritic score data, we performed a two-way ANOVA to examine the differences among all groups. We compared the differences between the treatment and cia-vehicle groups in other animal experiments using the Mann–Whitney *U* test. Statistical analyses were conducted using GraphPad Prism v8.0 software. Differences with a *P*-value of below 0.05 were considered statistically significant.

## 3 Results

### 3.1 Erianin ameliorated Th17 overactivation and suppressed IL-17 production via the JAK/STAT3 pathway

**Figure 1:**
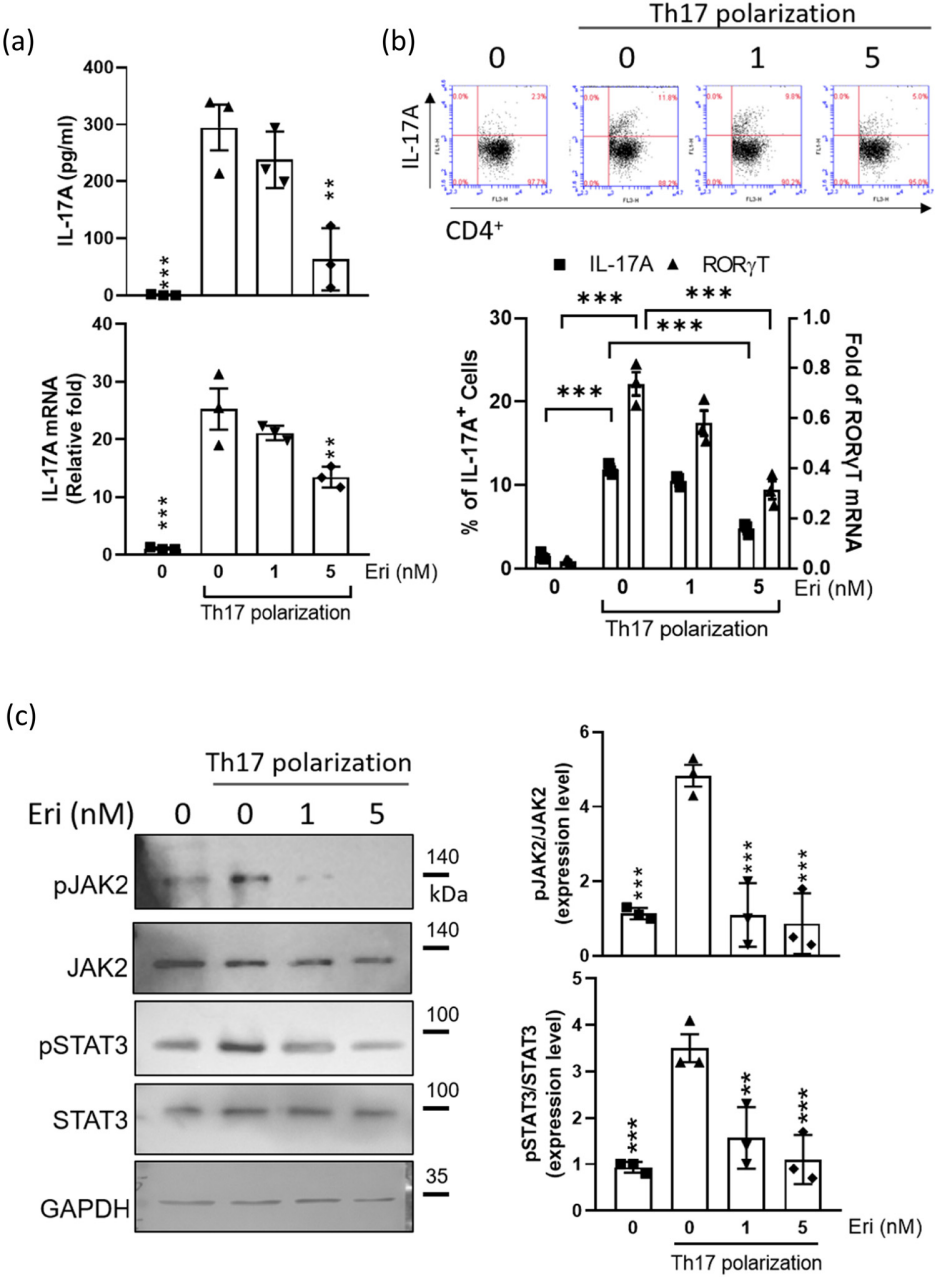
Effect of erianin on primary Th17 differentiation. (a) IL-17A protein concentrations in the culture medium and mRNA expression in cells were analyzed by ELISA and quantitative RT-PCR at 72 h posttreatment (hpt). The mRNA level is presented as the fold change relative to the untreated control (set as 1.0). (b) The percentage of the IL-17+ subpopulation and the RORγT mRNA level in CD4+ T cells were examined by flow cytometry and qRT-PCR, respectively, at 72 hpt. (c) Representative western blot of JAK/STAT pathway components and quantified protein expression normalized against the corresponding nonphosphorylated proteins by ImageJ software before plotting as bar graphs. Data are presented as the mean ± SEM of three wells from one of three experiments, where (***) *P* < 0.001 indicates significance compared with CD4+ T cells under Th17-polarizing conditions without erianin treatment (0 nM) via one-way ANOVA.

We first isolated CD4+ T cells from DBA/1 mice, one of the CIA-prone mouse strains [18], and treated them with anti-CD3 and anti-CD28 antibodies and the cytokines IL-6 and TGF-β to induce the differentiation of Th17 cells. The IL-17 mRNA transcripts and IL-17A levels, produced or released by Th17 cells, were used to evaluate Th17 polarization. Our results in Figure 1a and b indicate that the differentiation of primary CD4+ cells toward the Th17 subset was substantially stymied by erianin treatment at 5 nM or another concentration, with treated groups showing lower IL-17 transcript levels and IL-17A production. Consistently, treatment with the same concentration of erianin halted the increasing population of Th17 cells identified by staining for RORγt, Th17-specific transcripts, or intracellular IL-17A proteins, as determined by flow cytometry analysis. Our data revealed that a greater than 50% decrease in Th17 polarization of primary CD4+ T cells could be achieved with 5 nM erianin treatment (Figure 1c), indicating the remarkable effects of erianin in ameliorating the overactivation of CD4+ T cells.

To further confirm the suppressive capability of erianin, we examined the signaling pathway involved in Th17 development, IL-6-mediated JAK2/STAT3 signaling [19]. We focused on detecting changes in the phosphorylation of JAK and STAT3 (pJAK2, Tyr1007, pSTAT3, and Tyr705) with or without erianin treatment to investigate pathway activation. As shown in Figure 1, the phosphorylation of JAK and STAT3 elicited by Th-17 polarization was dose-dependently inhibited upon erianin treatment; the quantified data were in line with the western blotting images, with the ratios of phosphorylated protein to the cytoskeletal protein being significantly reduced. The data suggest that the downregulation of JAK2/STAT3 pathway activity appears to be one of the canonical mechanisms that inhibit the Th17 polarization of splenic CD4+ T cells.

**Figure 2:**
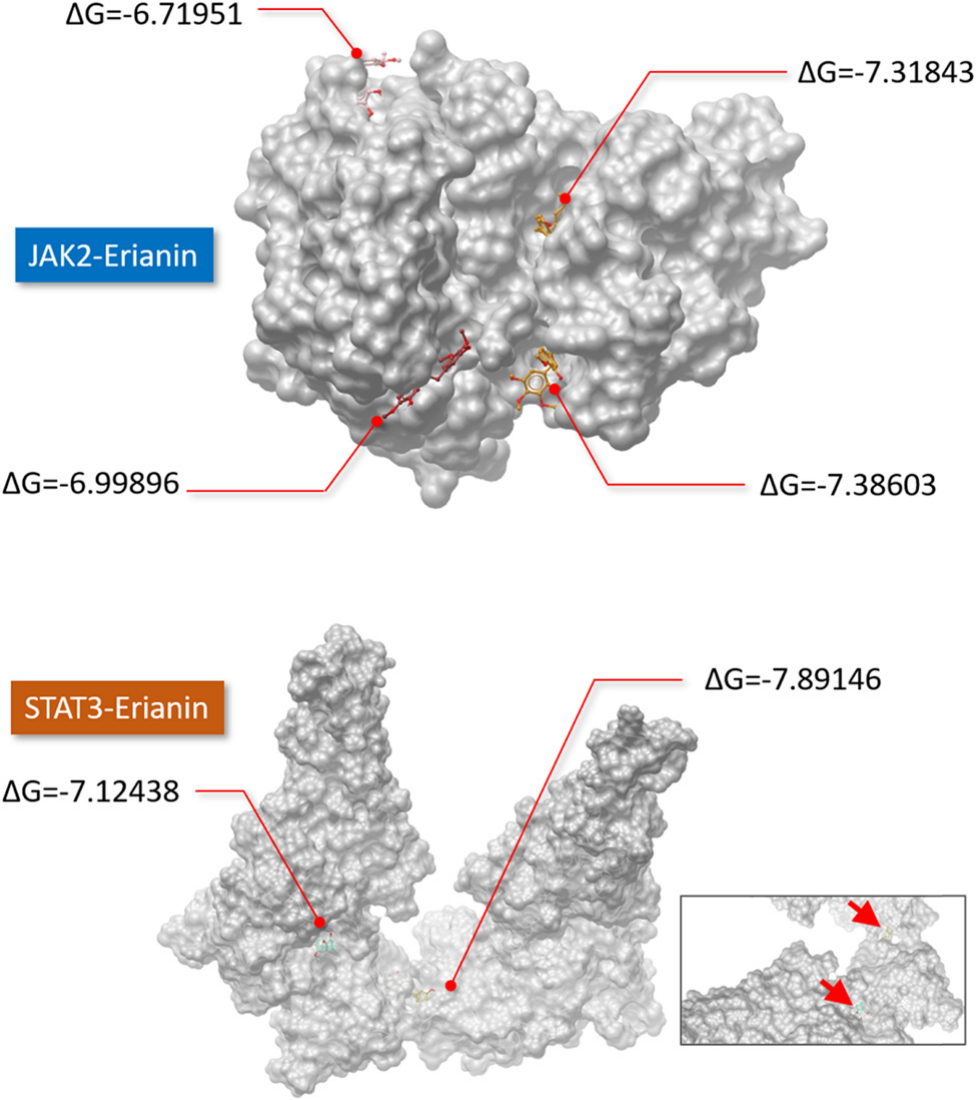
Molecular docking models for JAK2 or STAT3 with erianin. The representative docking simulations of erianin with JAK2 (above panel) or STAT3 (below panel), including all significant docking pockets, were visualized with the ICM browser in a space-filling model along with the indications of Δ*G* values. A lower Δ*G* value suggests stronger binding between two given molecules. The docking simulation was performed by SwissDock.

We performed molecular docking simulations between erianin and JAK2 or STAT3 to visualize the possible interacting locations where erianin might engage and interfere with phosphorylation. The images in Figure 2 display representative models. We found more docking pockets for erianin in JAK2 than in STAT3. Nevertheless, the values of Δ*G* between erianin and STAT3 were lower on average (Figure 2), suggesting that the molecular binding of erianin to STAT3 could be more stable.

### 3.2 Erianin alleviated arthritic manifestations *in vivo*

**Figure 3:**
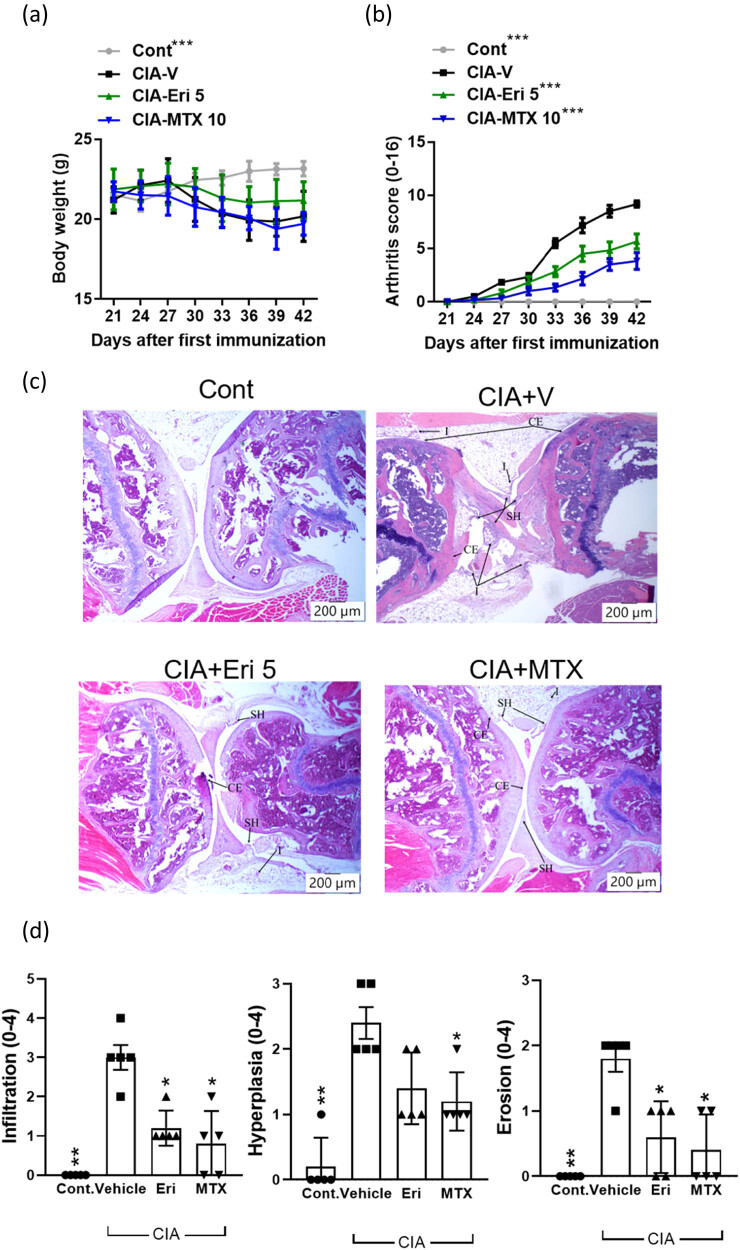
Antiarthritic effect of erianin in collagen-induced arthritis mice. CIA mice were given erianin (5 mg/kg), MTX (10 mg/kg), or vehicle treatment intragastrically (*N* = 5), followed by monitoring of (a) body weight and (b) clinical scores for arthritis from days 21 to 42. Significance is labeled as (***) *P* < 0.001 versus the CIA vehicle control group after analyses with two-way ANOVA. (c) Representative images of histopathology evaluated by H&E staining (100× magnification) on day 42. (d) Pathological scores were assessed for inflammatory cell infiltration, synovial hyperplasia, and bone erosion/cartilage damage. Data are presented as the mean ± SEM from two independent experiments (*N* = 5 or more). Significance is labeled as (*) *P* < 0.05 and (**) *P* < 0.01 versus the CIA vehicle control group after analyses with the Mann–Whitney *U* test.

As mentioned earlier, we established a mouse model using DBA/1 mice induced to develop arthritis by administering collagen, and Th17 cells are crucial to the pathogenic development in the CIA model. With this model, we examined and compared the therapeutic effects of erianin (5 mg/kg) and the current RA drug MTX (10 mg/kg) after the onset of CIA. We found that despite no significant improvement in weight (Figure 3a), mice treated with erianin exhibited a lower arthritic score than vehicle-treated mice, with the score being comparable to that of MTX-treated mice (Figure 3b). In addition to the arthritic levels, the pathological indications in ankle joint tissues from mice that received erianin were substantially akin to those in tissues from naïve control mice, showing a complete joint structure and scarce infiltrated immune cells, which were similar to those in tissues from MTX-treated mice (Figure 3c). Additionally, remarkable therapeutic effects were demonstrated with improved synovial hyperplasia and less cartilage damage (Figure 3d). Overall, the amelioration of arthritic manifestations and prevention of structural deterioration by erianin in CIA mice suggest that erianin likely possesses remarkable antiarthritic activity. With these positive results, we next aimed to elucidate whether erianin is involved in the modulation of Th17 activity in the CIA model.

### 3.3 Erianin suppressed Th17 cell expansion in the spleen

**Figure 4:**
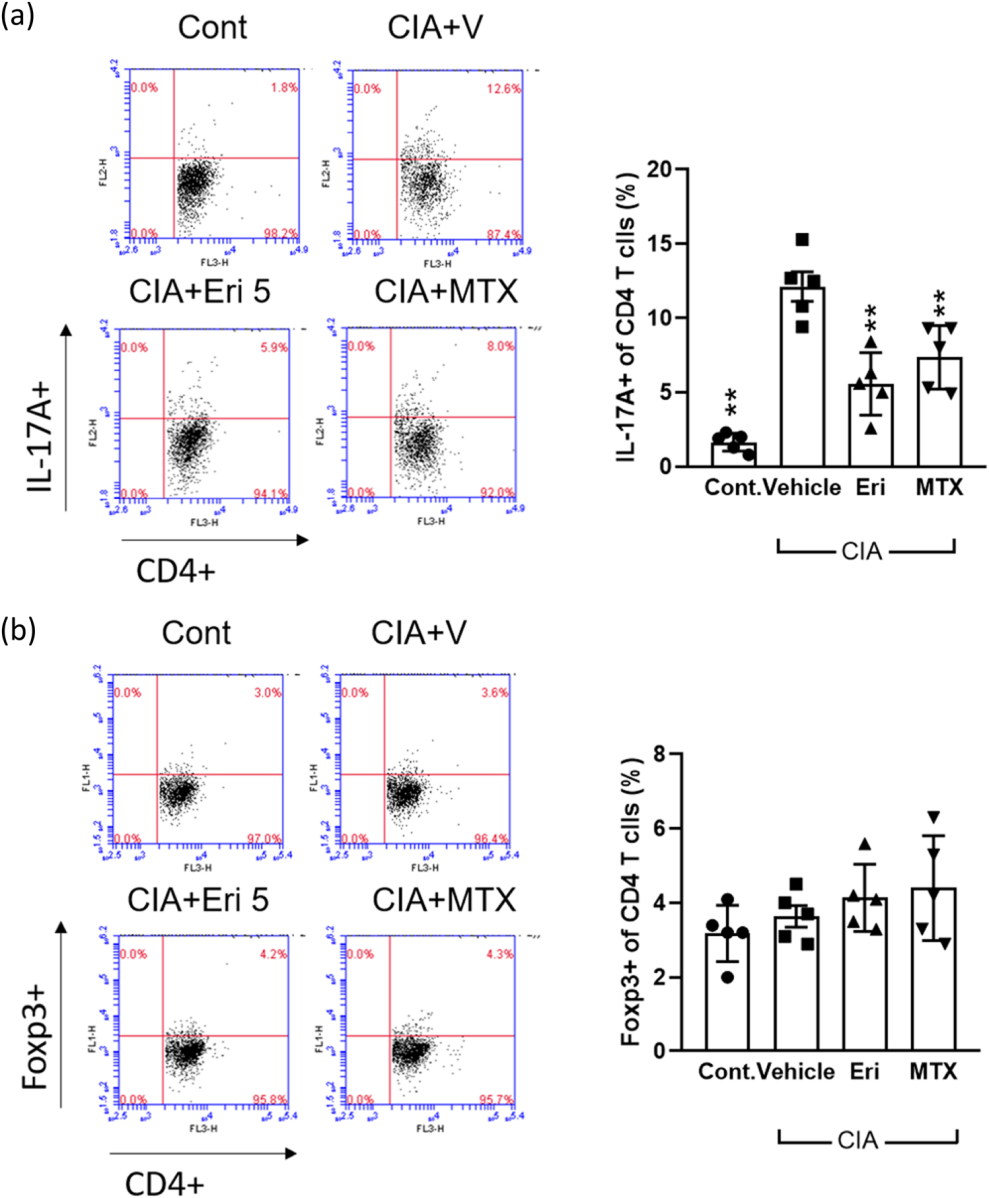
Effects of erianin on Th17 cell expansion *in vivo*. CIA mouse spleens were collected and analyzed. Splenocytes (2  ×  10^6^/ml) were cultured with bovine CII (20 μg/ml) for 72 h, stained with anti-CD4 and anti-IL-17A or anti-Foxp3 Abs, and analyzed by flow cytometry. Flow cytometric dot plots of intracellular staining for (a) IL-17A and (b) Foxp3 in T cells obtained from the spleen of one representative mouse from each group. Bar graphs are presented as the mean ± SEM from two independent experiments (*N* = 5 or more). Significance is labeled (**) *P* < 0.01 versus the CIA vehicle control group after analyses with the Mann–Whitney *U* test.

As soon as erianin treatment for 21 days was completed, we harvested the spleen of CIA mice and stimulated splenocytes with collagen type II (CII) *ex vivo* for an additional 72 h, followed by flow cytometry analyses to estimate T-cell subpopulations, especially IL-17A + CD4+ Th17 and FOXP3 + CD4+ regulatory T cells (Tregs). Our data in Figure 4 indicate that splenocytes from CIA mice were more apt to polarize and expand as Th17 cells than Tregs. However, splenocytes from either erianin- or MTX-treated mice showed a reduced tendency to differentiate into Th17 cells (Figure 4a) with slight but nonsignificant increases in Treg populations (Figure 4b), arguing that the activation of an aberrant T-cell response was suppressed following erianin administration.

### 3.4 Erianin decreased the levels of inflammatory mediators in paw tissues

**Figure 5:**
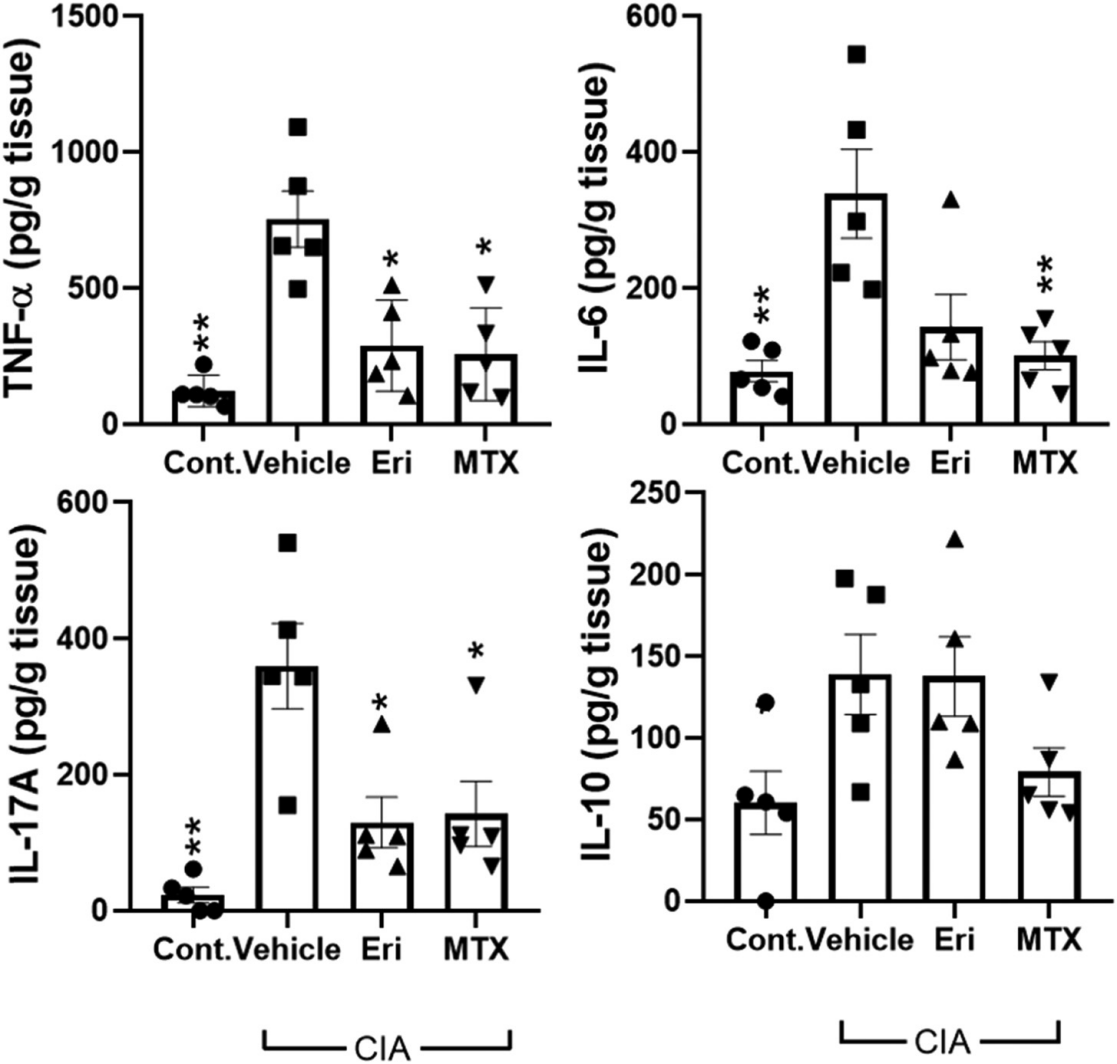
Effects of erianin on cytokine production in CIA mice. The paw joints of erianin-, MTX-, and vehicle-treated mice were collected to measure the levels of the proinflammatory cytokines IL-6, TNF-α, and IL-17A and the anti-inflammatory cytokine IL-10 by ELISA. Bar graphs are presented as the mean ± SEM from two independent experiments (*N* = 5). *P  <  0.05, **P  <  0.01 versus the CIA vehicle group, as determined by the Mann–Whitney *U* test.

Finally, we measured the concentrations of essential inflammatory or anti-inflammatory cytokines, including TNF-α, IL-6, IL-17A, and IL-10, in the paw tissues of CIA mice that received erianin, MTX, or vehicle treatment. The ELISA data shown in Figure 5 consistently revealed that the levels of TNF-α and IL-17 cytokines were profoundly decreased in the erianin-treated mice, with treatment reducing the levels of these cytokines to levels comparable to those in naïve control and MTX-treated mice. Despite no significant alteration in the IL-10 concentration, the ELISA data indicated that the inflammatory condition in CIA joints was alleviated after erianin treatment, supporting the overall therapeutic effect of erianin against CIA.

## 4 Discussion

RA is a complex autoimmune disorder, as patients can develop aberrant symptoms that keep them from being capable of performing daily activities, continuously reducing their quality of life for years. Due to the complexity of RA pathogenesis, only some remedies will benefit all patients. The most widely adopted drugs for treating RA are disease-modifying antirheumatic drugs (DMARDs), including conventional, biological, and targeted synthetic DMARDs. Nevertheless, the efficacy of these drugs, such as MTX, can produce a response rate varying from 30 to 60% even with combined treatments [20]. The effectiveness of erianin against CIA demonstrated in the present study provides an alternative remedy to be considered a monotherapy or combination therapeutic strategy to enhance patient outcomes. In our CIA model, erianin produced an effect comparable to MTX treatment. Notably, erianin-treated mice showed less weight loss than MTX-treated mice, suggesting that erianin might improve food intake or appetite (Figure 3a).

Regarding the mechanisms of erianin, our data indicated that suppressing overwhelming Th17 activation appeared to be the most likely pathway by which erianin alleviated all CIA symptoms (Figures 1 and 3). However, no effect was associated with an upregulation of Tregs as essential as Th17 cells in CIA pathogenesis. Treg dysfunction can also cause disease progression in arthritis [21]. Despite the intricate functions of Tregs, it has been shown that providing Tregs exogenously to CIA mice can ameliorate CIA by decreasing the Th1/Th2 ratio and B-cell population [22]. However, erianin treatment seemed unrelated to regulating Treg numbers or activities. Interestingly, MTX treatment was demonstrated to restore defective regulatory T cells [23]. This information could shed light on the combined use of erianin to treat RA patients with a poor response to MTX.

Regarding the inhibitory effect of erianin on the phosphorylation of JAK2 and STAT3 (Figure 1c), we noticed that the inhibitory effect was enhanced when the concentration of erianin was increased. However, the phosphorylation of JAK2 and STAT3 slightly differ in response to increasing erianin concentrations. Despite the anti-phosphorylation effect of a high concentration of erianin, the phosphorylation of STAT3, but not that of JAK2, was also inhibited by erianin at a concentration as low as 1 nM. Our molecular docking modeling might provide a clue to explain the difference; the Δ*G* for the erianin and STAT3 docking model was lower than that for the erianin–JAK2 interaction (Figure 2). A lower Δ*G* indicates stronger binding between two given molecules [24]. As a result, erianin might be more effective in inhibiting the phosphorylation of STAT3.

## 5 Conclusion

**Figure 6:**
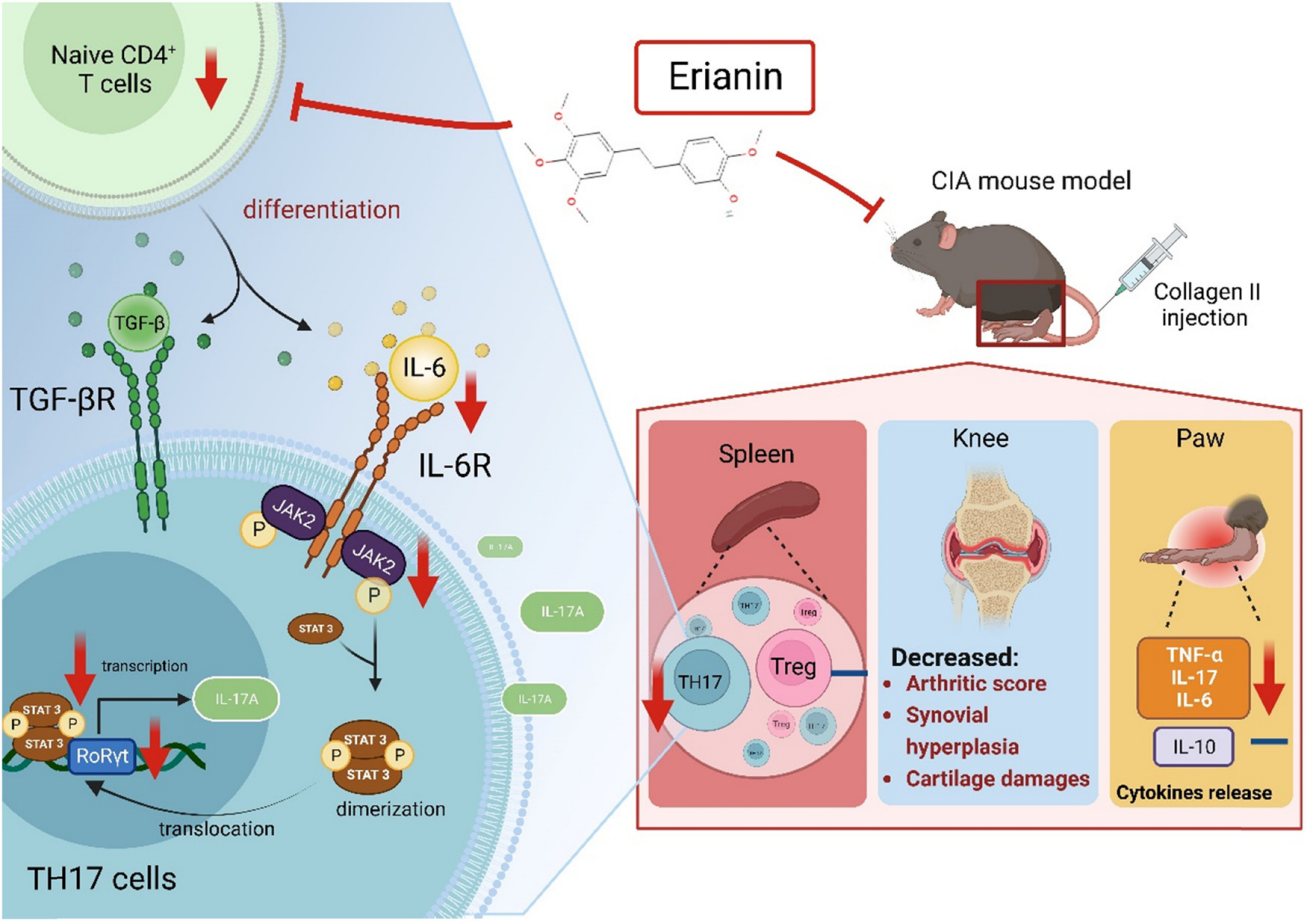
Proposed model of the mechanisms of erianin that alleviate CIA in the mouse model.

Collectively, our current study demonstrated the antiarthritic potency of erianin *in vitro* and *in vivo*. Pathogenic Th17 polarization and its cellular differentiation pathway were consistently suppressed by erianin treatment. All of these factors synergistically contribute to the severity of arthritis in mice, as graphically summarized in Figure 6. CIA mice that received erianin treatment exhibited a better outcome and recovery, supporting the pharmaceutical potential of erianin. The therapeutic effect of erianin was comparable to that of MTX, which could soon lead to the development of an alternative or combinational treatment for RA.
